# Investigation of the Microstructure and Scintillation Properties of Ce-Doped CaF_2_/LiF Eutectics for Thermal Neutron Detection

**DOI:** 10.3390/ma19061102

**Published:** 2026-03-12

**Authors:** Tomoaki Matsuyama, Kei Kamada, Masao Yoshino, Rikito Murakami, Satoshi Ishizawa, Yuui Yokota, Akira Yoshikawa

**Affiliations:** 1 Graduate School of Engineering, Tohoku University, 6-6 Aramaki Aza Aoba, Aoba-ku, Sendai 980-8579, Miyagi, Japan; 2 Institute for Materials Research, Tohoku University, 2-1-1 Katahira, Aoba-ku, Sendai 980-8577, Miyagi, Japanrikito.murakami.d4@tohoku.ac.jp (R.M.); satoshi.ishizawa.a2@tohoku.ac.jp (S.I.); yui.yokota.a5@tohoku.ac.jp (Y.Y.); akira.yoshikawa.d8@tohoku.ac.jp (A.Y.); 3 C&A Corporation, 1-16-23 Ichibancho, Aoba-ku, Sendai 980-0811, Miyagi, Japan; 4 New Industry Creation Hatchery Center, Tohoku University, 6-6-10 Aramaki, Aoba-ku, Sendai 980-8579, Miyagi, Japan

**Keywords:** ^6^Li, scintillator, neutron, eutectic, CaF_2_/LiF, melt growth, Ce^3+^ 5d–4f, pulse-height discrimination

## Abstract

With the growing global emphasis on nuclear reactor decommissioning, reliable thermal neutron detection has become increasingly important for ensuring critical safety and for the identification of fuel debris and radioactive waste. In this context, this study developed and characterized a Ce-doped CaF_2_/^6^LiF (Ce:CaF_2_/LiF) eutectic scintillator for thermal neutron detection with Ce concentrations ranging from 0.5 to 10 mol%. The eutectic samples were grown by the melt-solidification method, and their crystalline properties were evaluated using inductively coupled plasma mass spectrometry, X-ray diffraction, scanning electron microscopy, and field-emission electron probe microanalysis. Radioluminescence, photoluminescence, transmittance, scintillation decay, and pulse-height measurements were conducted to assess their scintillation performance. Structural characterization revealed a well-defined eutectic microstructure together with several Ce-rich phases. The results of the effective neutron sensitivity demonstrated that the Ce concentration was effectively optimized based on the effective neutron sensitivity: the sample with 1 mol% Ce exhibited the highest neutron sensitivity (approximately 1.5 times that of a Ce:LiCaAlF_6_ single crystal) and a 1.6-times higher neutron-induced light yield, while maintaining a fast effective decay time of 400 ns. These findings suggest that the Ce:CaF_2_/LiF eutectic is a promising candidate for high-performance thermal-neutron scintillators for applications in nuclear decommissioning.

## 1. Introduction

Global emphasis on nuclear reactor decommissioning has recently increased significantly [1,2]. In this process, accurate neutron detection plays a crucial role in managing the risk of recriticality and in identifying and classifying fuel debris, radioactive waste, and related materials. In practice, however, neutron detection is particularly challenging—when only small amounts of fissile material are present, the neutron emission rate is low, whereas the γ-ray background can exceed ~10 Gy h^−1^ due to strong radioactive contamination [3,4,5].

In nuclear decommissioning applications, Ce:LiCaAlF_6_ (Ce:LiCAF) has been widely recognized as a promising scintillator owing to its fast decay time (30–50 ns), the large neutron absorption cross-section of ^6^Li (940 barn), the high Q-value (4.78 MeV), low density (2.94 gcm^−3^), excellent n/γ discrimination capability, and outstanding chemical stability with non-hygroscopicity [6,7,8,9,10]. Building on these advantages, our group previously compared ultra-thick (~100 µm) Ce:LiCAF with conventional Li glass (KG2) under high γ-dose-rate conditions and evaluated n/γ discrimination by pulse-height discrimination (PHD) [11]. Ce:LiCAF was observed to maintain a stable effective neutron count rate (n_eff_) up to 2.97 Gy h^−1^ in intense γ-ray fields, whereas KG2 exhibited a decrease of approximately 20% in n_eff_. Nevertheless, practical implementation requires further enhancements, including (i) increasing the ^6^Li concentration to improve neutron sensitivity, (ii) shortening the decay time to enable high-rate radiation measurements, and (iii) reducing γ-ray sensitivity by decreasing the scintillator thickness or density.

Our group has long pursued the development of ^6^Li-containing eutectic scintillators for thermal neutron detection [12,13,14,15]. In these eutectic scintillators, a neutron-capture phase converts incident neutrons via the ^6^Li(n,α)^3^H reaction:

(1) Li6+n→α2.05 MeV+H32.73 MeV   Q=4.78 MeV.

The resulting α-particles and tritons are subsequently absorbed by the scintillation phase, which primarily emits visible light. Unlike single crystals—whose ^6^Li concentration is limited by stoichiometry—eutectic microstructures allow for higher lithium content. Moreover, eutectic scintillator fabrication is simpler and more productive than conventional single-crystal growth, making them highly attractive from an industrial perspective.

At Ce concentrations less than 10 mol%, Ce:CaF_2_ single crystals exhibit high α-induced light yields (1000–7000 ph/5.5 MeV) and low γ-ray light yields (800–950 ph/MeV), suggesting a superior α/γ ratio compared with Ce:LiCAF single crystals [16]. The X-ray induced scintillation decay time is relatively fast, ranging from approximately 40 to 250 ns [16]. At the CaF_2_/LiF eutectic composition, the theoretical Li concentration reaches 0.063 mol cm^−3^—approximately four times higher than that of Ce:LiCAF—while the density remains lower (~2.8 g cm^−3^ versus 3.0 g cm^−3^ for Ce:LiCAF). Moreover, the eutectic CaF_2_/LiF system exhibits nearly identical volumetric fractions of the two phases (46:54) and a relatively small refractive-index mismatch between them. As a result, the eutectic system is expected to exhibit inherently high neutron response, reduced γ-ray sensitivity, and efficient energy transfer between the neutron-capture and scintillation phases.

Therefore, in this study, Ce:CaF_2_/LiF eutectic scintillators with Ce concentrations ranging from 0.5 to 10 mol% were fabricated, and their microstructural features, scintillation properties, and neutron sensitivity based on PHD were systematically investigated and compared with those of conventional Ce:LiCAF scintillators. In this eutectic system, ^6^LiF functions as the neutron-capture phase, while Ce:CaF_2_ acts as the scintillation phase.

## 2. Materials and Methods

### 2.1. Eutectic Growth

The eutectics were grown by melt-solidification method. High-purity (4 N) powders of 95% enriched ^6^LiF, CaF_2_, and CeF_3_ were used as starting materials. The ^6^LiF and CaF_2_ powders were mixed at a molar ratio of 80.5:19.5, corresponding to the eutectic composition [17], and CeF_3_ was added at 0.5, 1, 3, 5, 7, and 10 mol% with respect to CaF_2_. Approximately 3 g of the mixed powder was loaded into a carbon crucible (inner diameter: 14 mm, height: 20 mm). The mixture was preheated at approximately 200 °C under a 10^−4^ Pa vacuum for 6 h to remove residual moisture. Subsequently, the atmosphere was replaced with an Ar–CF_4_ gas mixture (8:2 ratio). The carbon heater used high-frequency induction heating to melt the raw materials. After maintaining the high-frequency power for 30 min, the power was immediately shut down to solidify the melt. The obtained eutectic ingots were cut and polished along the cross-sectional plane using a wire saw. A Ce:LiCAF single crystal (10 mm × 10 mm × 0.5 mm, 3 mol% Ce, and 95% ^6^Li, CZ-grown; C&A Corp., Sendai, Japan) was used as a reference. Crystalline phases and scintillation properties were characterized using the methods described in the following sections.

### 2.2. Phase and Microstructural Characterization

For compositional analysis, measurement samples were extracted from the top, middle, and bottom positions of the tablet-shaped eutectic along the vertical direction and ground into fine powder for measurement. Inductively coupled plasma mass spectrometry (ICP-MS, Agilent 8800, Agilent Technologies, Santa Clara, CA, USA) was performed to determine the composition variation along the solidification direction. Phase identification was conducted by X-ray diffraction (XRD) using a D8 DISCOVER diffractometer (Bruker, Billerica, MA, USA). Measurements were conducted over a 2θ range of 20–60° using Cu Kα radiation operated at 40 kV and 40 mA. Microstructural observations and evaluations were performed using backscattered electron (BSE) imaging and field-emission electron probe microanalysis (FE-EPMA; JXA-8530F, JEOL Ltd., Tokyo, Japan).

### 2.3. Optical and Scintillation Measurements

Transmittance spectra were measured using a UV/vis spectrophotometer (V-770, JASCO, Tokyo, Japan) in the wavelength range of 190–800 nm. The photoluminescence (PL) emission and PL excitation (PLE) spectra were measured using a spectrometer (FLS 1000, Edinburgh Instruments, Livingston, UK) with a Xe lamp. Radioluminescence (RL) spectra were measured under excitation by an Ag-target X-ray tube operated at 40 kV and 40 mA. The emitted light was analyzed using a charge-coupled device (CCD) camera (iDus420-OE, Andor, Belfast, UK) coupled with a spectrometer (SR-163, Andor). Light yields of the eutectic samples and a Ce:LiCAF single-crystal reference with comparable volume and thickness were evaluated under ^252^Cf neutron irradiation. Each sample was coupled to a photomultiplier tube (PMT; R7600-200, Hamamatsu, Iwata City, Japan) using optical grease (KF-96H-60000CS, Shin-Etsu Chemical, Tokyo, Japan). The PMT was operated at 700 V. For neutron measurements, a 5 cm-thick polyethylene moderator and a 5 cm-thick lead block for γ-ray shielding were placed between the ^252^Cf source and the sample, with a source–sample distance of approximately 15 cm. For γ-ray measurements, the ^60^Co source was positioned about 5 cm from the sample without a moderator. Pulse-height spectra were recorded using a two-channel universal serial bus (USB) wave catcher module [18] with a shaping time of 2 µs. The corrected count rate ($n_{c}$) was calculated as:

(2) $$n_{c}=\frac{n_{s}}{1-R_{d}},$$

where $n_{s}$ is the throughput rate and $R_{d}$ is the dead-time ratio, defined as $T_{d}/T_{r}$ (dead time/real time). The spectra were fitted using a Gaussian function as:

(3) $$Fit\left(C\right)=Aexp\left\{{-\left(\frac{C-\mu }{\sqrt{2}\sigma }\right)}^{2}\right\},$$

where A, μ, and σ are the height, mean, and standard deviation, respectively.

The effective neutron count rate ($n_{eff}$) was obtained by integrating the Gaussian fit above the discrimination threshold $C_{th}$ as:

(4) $$n_{eff}=\int_{C_{th}}^{\infty }Fit\left(C\right)dC=A\sigma \sqrt{\frac{\pi }{2}}\left\{1-erf\left(\frac{C_{th}-\mu }{\sqrt{2}\sigma }\right)\right\},$$

where $C_{th}$ represents the threshold channel for neutron–γ discrimination, defined as the channel corresponding to the minimum between the γ-ray continuum and the neutron-induced peak in the pulse-height spectrum containing both neutron and γ-ray signals. This threshold enables the most effective separation of γ-ray-induced events from neutron-induced events. Decay-time measurements were performed by directly connecting the PMT to an oscilloscope (TDS3035B, Tektronix, Beaverton, OR, USA).

Each decay curve, obtained by averaging 120 waveforms, was fitted with a multi-exponential function as:

(5) $$I\left(t\right)=\sum_{i}I_{i}\;exp\left(-\frac{t}{\tau_{i}}\right)+B,$$

where I(t), $I_{i},$ t, $\tau_{i},$ and B denote the luminescence intensity, initial intensity, time, decay constant, and background, respectively. The effective decay time ($\tau_{eff}$) was calculated as:

(6) $$\tau_{eff}=\frac{\sum_{i}I_{i}{\tau_{i}}^{2}}{\sum_{i}I_{i}\tau_{i}}.$$

## 3. Results

### 3.1. Eutectics

**Figure 1:**
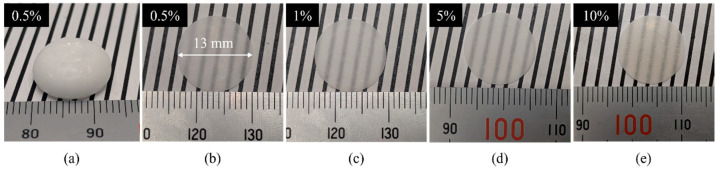
Photographs of the as-grown 0.5 mol% Ce:CaF_2_/LiF eutectic (**a**) and approximately 0.47 mm-thick wafers prepared from the eutectics with different Ce-doping concentrations: (**b**) 0.5 mol%, (**c**) 1 mol%, (**d**) 5 mol%, and (**e**) 10 mol%.

The 0.5, 1, 3, 5, 7, and 10 mol% Ce-doped CaF_2_/LiF eutectics were fabricated by the melt-solidification method using the carbon crucible. Columnar-like, white, opaque as-grown eutectic ingots were obtained, as shown in Figure 1a, while Figure 1b–e display representative wafers prepared from the eutectics with various Ce concentrations. The average wafer thicknesses, determined as the mean of five measurement points on each wafer, were 0.49 ± 0.06, 0.48 ± 0.02, 0.47 ± 0.01, 0.47 ± 0.06, 0.46 ± 0.01, and 0.47 ± 0.04 mm for samples with 0.5, 1, 3, 5, 7, and 10 mol% Ce, respectively. As shown in the figure, optical transparency was successfully achieved across a wide range of Ce concentrations.

### 3.2. Phase and Microstructure 

**Figure 2:**
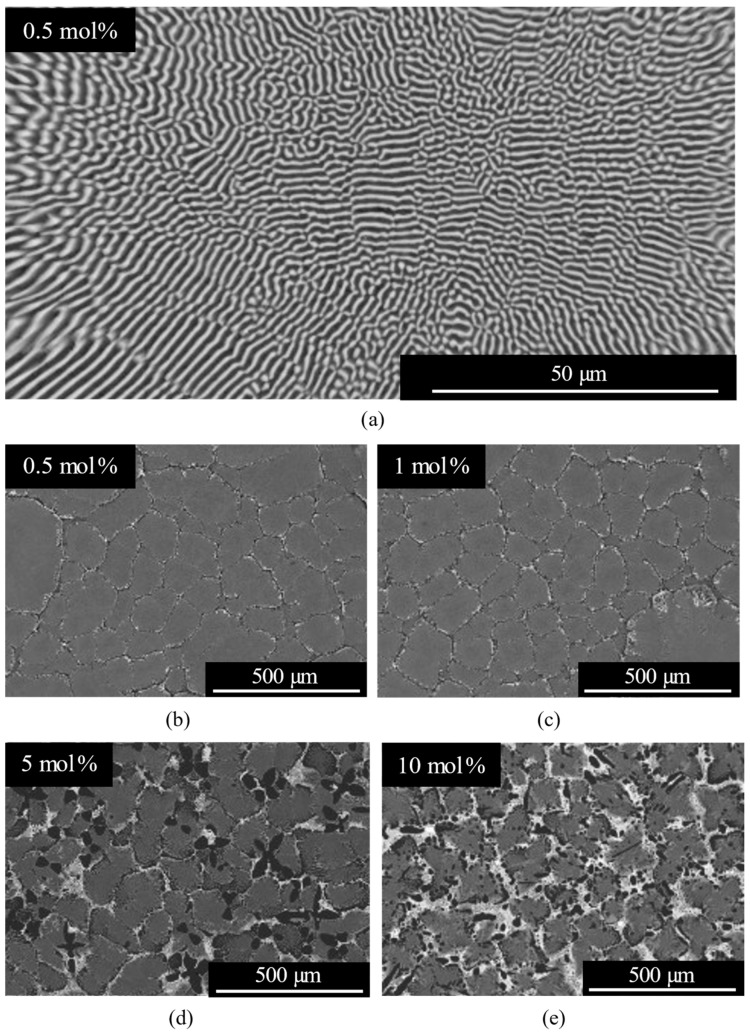
(**a**) Magnified BSE image of the CaF_2_/LiF eutectic region in the 0.5 mol% Ce-doped sample, and wider-area BSE images of the wafer cross sections prepared from the (**b**) 0.5 mol%, (**c**) 1 mol%, (**d**) 5 mol%, and (**e**) 10 mol% Ce-doped eutectics.

Figure 2 shows cross-sectional BSE images of wafers with different Ce concentrations. Figure 2a presents a high-magnification image of the 0.5 mol% Ce-doped sample, in which a fine LiF/CaF_2_ lamellar eutectic structure with an interlamellar spacing of ~1 µm was observed. As shown in Figure 2b–e, phases composed of Ce-rich elements were identified along the eutectic colony boundaries. The volume fraction of these Ce-rich phases increased with the nominal Ce concentration, ranging from 0.5 mol% to 10 mol%.

**Figure 3:**
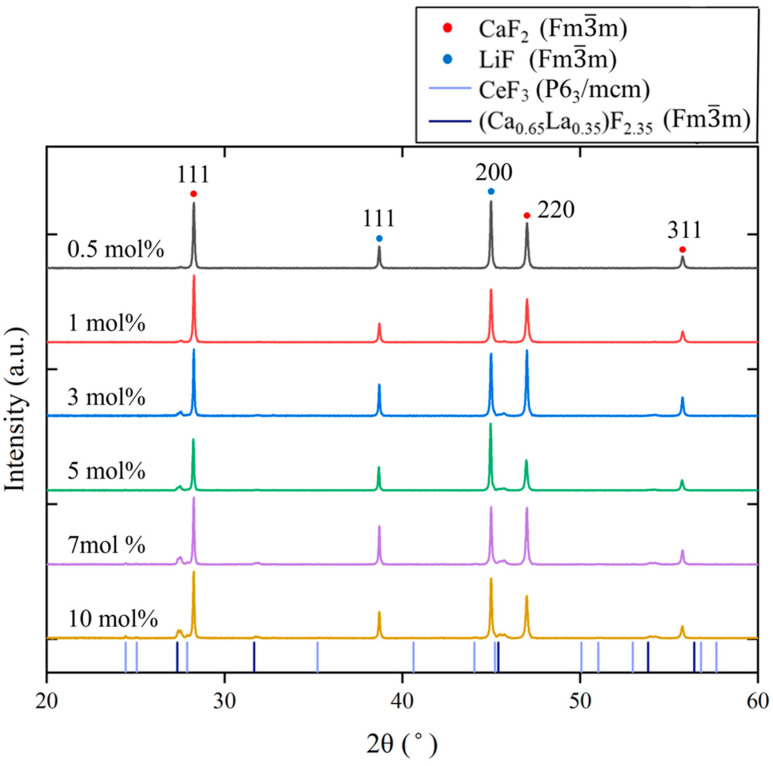
Powder XRD patterns of the Ce:CaF_2_/LiF eutectic samples with 0.5, 1, 3, 5, 7, and 10 mol% Ce concentrations. The colored drop lines indicate the diffraction peak positions of CeF_3_ and (Ca_0.65_La_0.35_)F_2.35_.

Figure 3 shows the powder XRD patterns of the Ce-doped eutectics. With increasing Ce concentration, in addition to reflections from CaF_2_ (Fm$\bar{3}$m, PDF 89-4794) and LiF (Fm$\bar{3}$m, PDF 04-0857), additional peaks corresponding to CeF_3_ (P6_3_/mcm, PDF 08-0045) appeared. Furthermore, diffraction peaks exhibiting a similar pattern to (Ca_0.65_La_0.35_)F_2.35_ (Fm$\bar{3}$m, PDF 87-0975) were also observed. La and Ce are adjacent in the periodic table and possess closely related chemical properties; therefore, Ce^3+^ ions are expected to behave similarly to La^3+^ in fluorite-related structures. Because the ionic radius of Ce^3+^ (1.14 Å) is slightly smaller than that of La^3+^ (1.16 Å), the diffraction peaks of the corresponding (Ca_0.65_Ce_0.35_)F_2.35_ or (Ca_x_,Ce_1−x_)F_3−x_ phases are expected to shift to higher angles compared with those of (Ca_0.65_La_0.35_)F_2.35_. This tendency was consistent with the observed XRD results.

**Figure 4:**
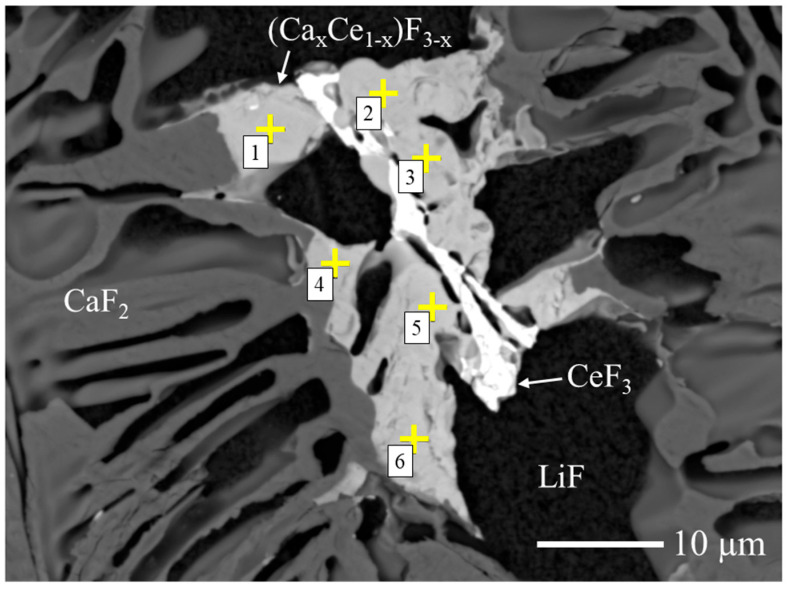
BSE image of the 10 mol% Ce-doped CaF_2_/LiF eutectic wafer used for FE-EPMA analysis. The phases identified by XRD are indicated, and the yellow “+” symbols with numbers (1–6) denote the six measurement points selected for compositional evaluation.

To estimate the Ca/Ce ratio in the fluorite-related (Ca_x_,Ce_1−x_)F_3−x_ phase, quantitative compositional analysis was performed by FE-EPMA. As shown in Figure 4, six measurement points were collected from the target phase in the 10 mol% Ce-doped sample, and the average composition was calculated. As summarized in Table 1, the average atomic ratio was Ca:Ce ≈ 68:32. Based on this result, the phase is hereafter referred to as (Ca_0.68_Ce_0.32_)F_2.32_, which represents the statistically averaged composition of the (Ca_x_,Ce_1−x_)F_3−x_ phase.

The ICP-MS results for the approximately 5 mol% Ce-doped eutectic are summarized in Table 2. The measurement samples were extracted from the top, middle, and bottom regions of the tablet-shaped 6 g as-grown eutectic along the vertical direction. Ce concentration showed an increasing trend toward the bottom of the eutectic. In the melt solidification method, the melt solidifies from the top toward the bottom. Such deviations could arise from a possible shift in the eutectic point of the ^6^LiF/CaF_2_ system relative to that of the natural LiF/CaF_2_ system, as well as from the effect of precipitation of the (Ca_0.68_Ce_0.32_)F_2.32_ and CeF_3_ phases.

### 3.3. Optical and Scintillation Properties

**Figure 5:**
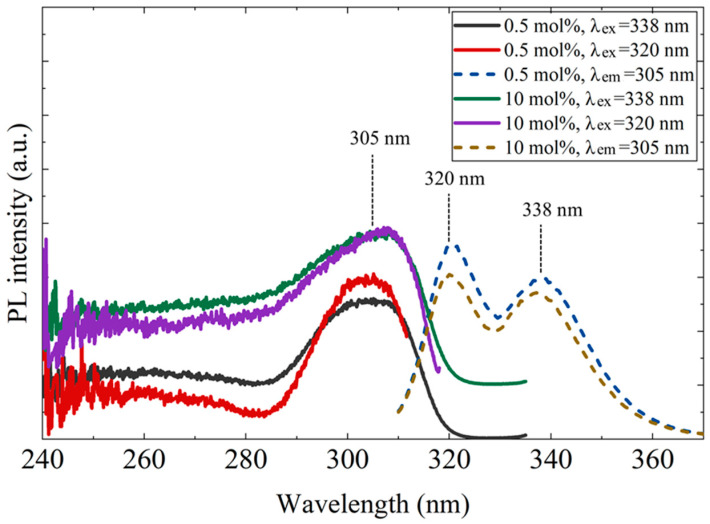
Photoluminescence excitation (PLE, left) and emission (PL, right) spectra of the 0.5 mol% and 10 mol% Ce-doped CaF_2_/LiF eutectic samples. In the inset, λ_ex_ and λ_em_ denote the excitation and emission wavelengths, respectively.

Figure 5 shows the photoluminescence (PL) and PL excitation (PLE) spectra of the 0.5 mol% and 10 mol% Ce-doped CaF_2_/LiF eutectics. Similar results were obtained for both samples. Upon excitation at 305 nm, distinct Ce^3+^ 5d–4f emission peaks appeared at approximately 320 and 338 nm. For both emission bands, a pronounced excitation peak was observed at around 305 nm, which was mainly attributed to the transition from the 4f^1^ ground state to the 5d^1^ excited state of Ce^3+^ associated with C_4_v (F^−^ vacancy–compensated) centers [19].

**Figure 6:**
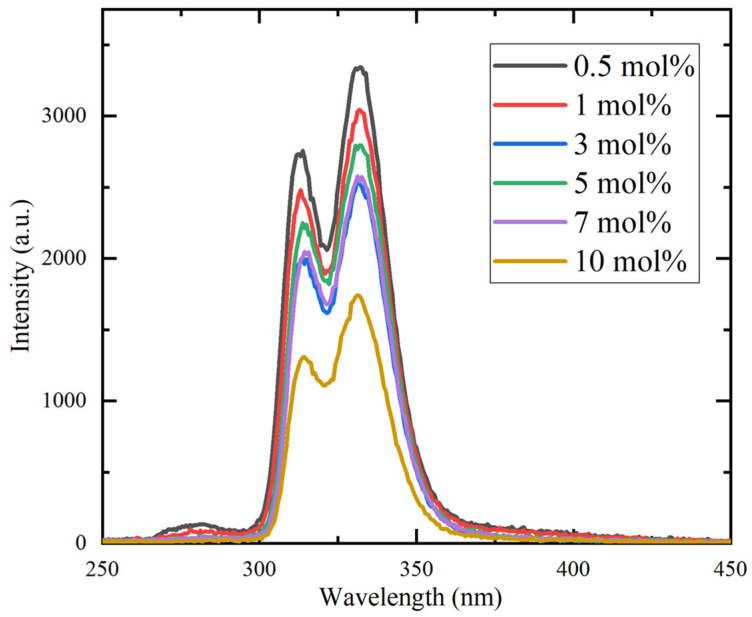
X-ray-induced radioluminescence spectra of the Ce:CaF_2_/LiF eutectic samples with 0.5, 1, 3, 5, 7, and 10 mol% Ce concentrations.

Figure 6 presents the RL spectra of the Ce:CaF_2_/LiF eutectics under Ag-target X-ray excitation. Two distinct emission peaks were observed in the 300–350 nm region, in good agreement with the PL results. In addition, a weaker emission band centered at approximately 280 nm was detected and assigned to self-trapped exciton (STE) luminescence [20,21]. The overall emission intensity decreased with increasing Ce concentration, which is in good agreement with the trend reported for Ce-doped CaF_2_ single crystals [16].

**Figure 7:**
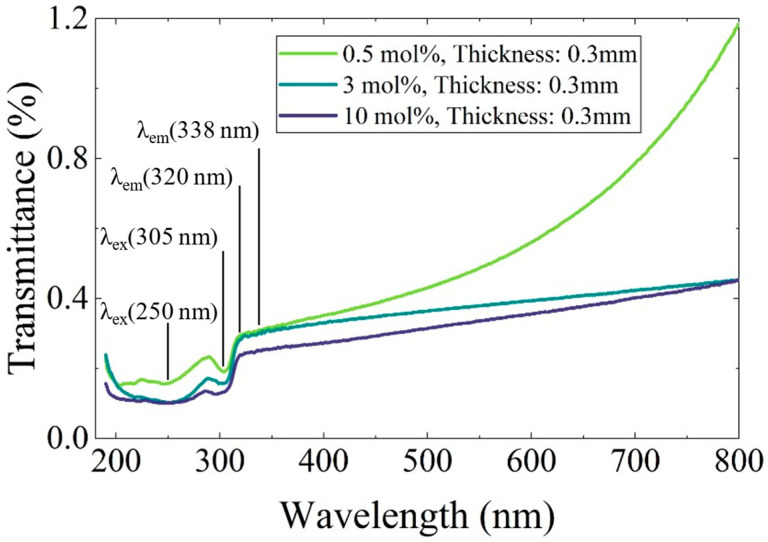
Transmittance spectra of Ce-doped CaF_2_/LiF eutectic samples with 0.5, 3, and 10 mol% Ce. The measurements were performed over the wavelength range of 190–800 nm.

To evaluate the influence of Ce-rich phases on the decrease in luminescence intensity, optical transmittance measurements were performed. Figure 7 presents the transmittance spectra of the 0.5, 3, and 10 mol% Ce-doped eutectic samples with comparable thickness. A distinct absorption band was observed around 305 nm, corresponding well to the PLE excitation band shown in Figure 5. In the 200–280 nm region, complex absorption features were observed, which are likely to originate from overlapping contributions of multiple defect-related centers, may include Li^+^-associated (around 250 nm) and oxygen-related defects (around 270 nm) [19]. Notably, no significant absorption was observed at 320 and 338 nm, corresponding to the Ce^3+^ 5d–4f emission wavelengths. This indicates that reabsorption of the emitted light by Ce-rich phases or other impurity-related levels is negligible in the present samples. Nevertheless, the overall transmittance decreases with increasing Ce concentration, primarily due to enhanced light scattering caused by the higher volume fraction of heavy Ce-rich precipitates, consistent with the microstructural features shown in Figure 2. Therefore, light scattering from the Ce-rich phases is considered one of the main factors contributing to the reduction in luminescence intensity at higher Ce concentrations.

**Figure 8:**
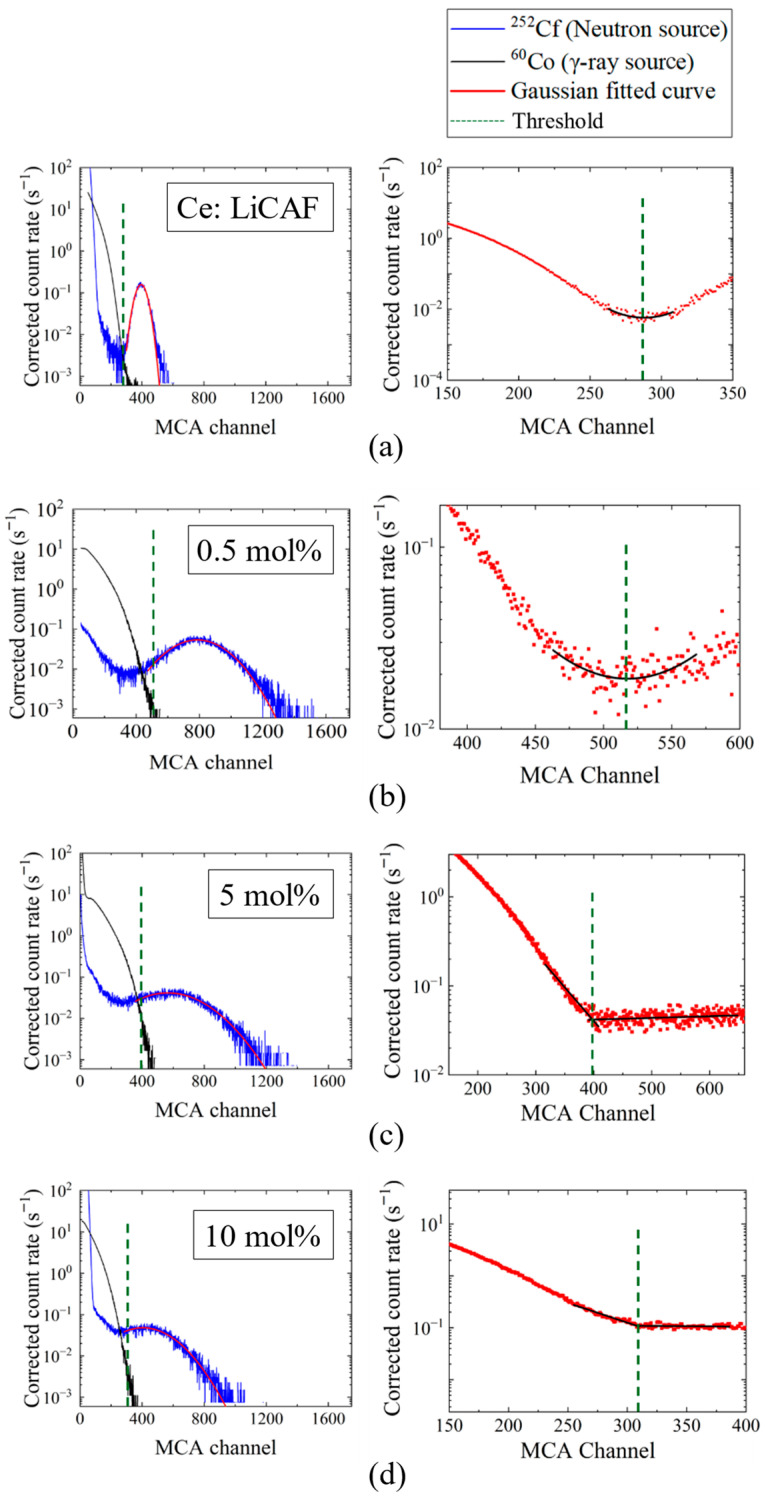
Pulse-height spectra (PHS) measured under ^252^Cf (neutron) and ^60^Co (γ-ray) irradiation for (**a**) the Ce:LiCAF standard and (**b**) 0.5 mol%, (**c**) 5 mol%, and (**d**) 10 mol% Ce-doped eutectic samples. For each sample, the left panel shows the individual PHS under ^252^Cf and ^60^Co irradiation together with the discrimination threshold, while the right panel presents the combined pulse-height spectrum of the ^252^Cf and ^60^Co signals used for threshold determination, along with the corresponding fitting curves.

**Figure 9:**
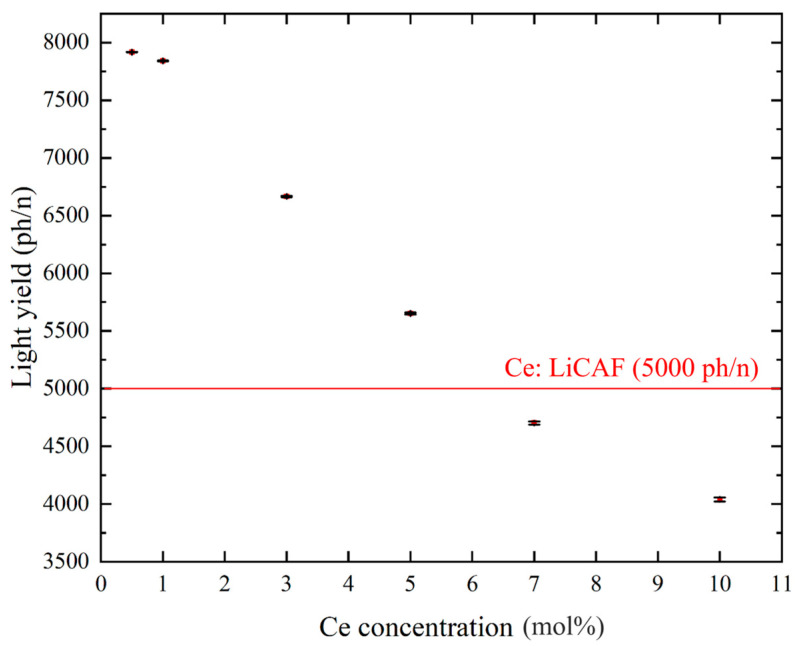
Scintillation light yield of Ce:CaF_2_/LiF eutectic crystals under ^252^Cf excitation, plotted as a function of Ce concentration (0.5, 1, 3, 5, 7, and 10 mol%).

**Figure 10:**
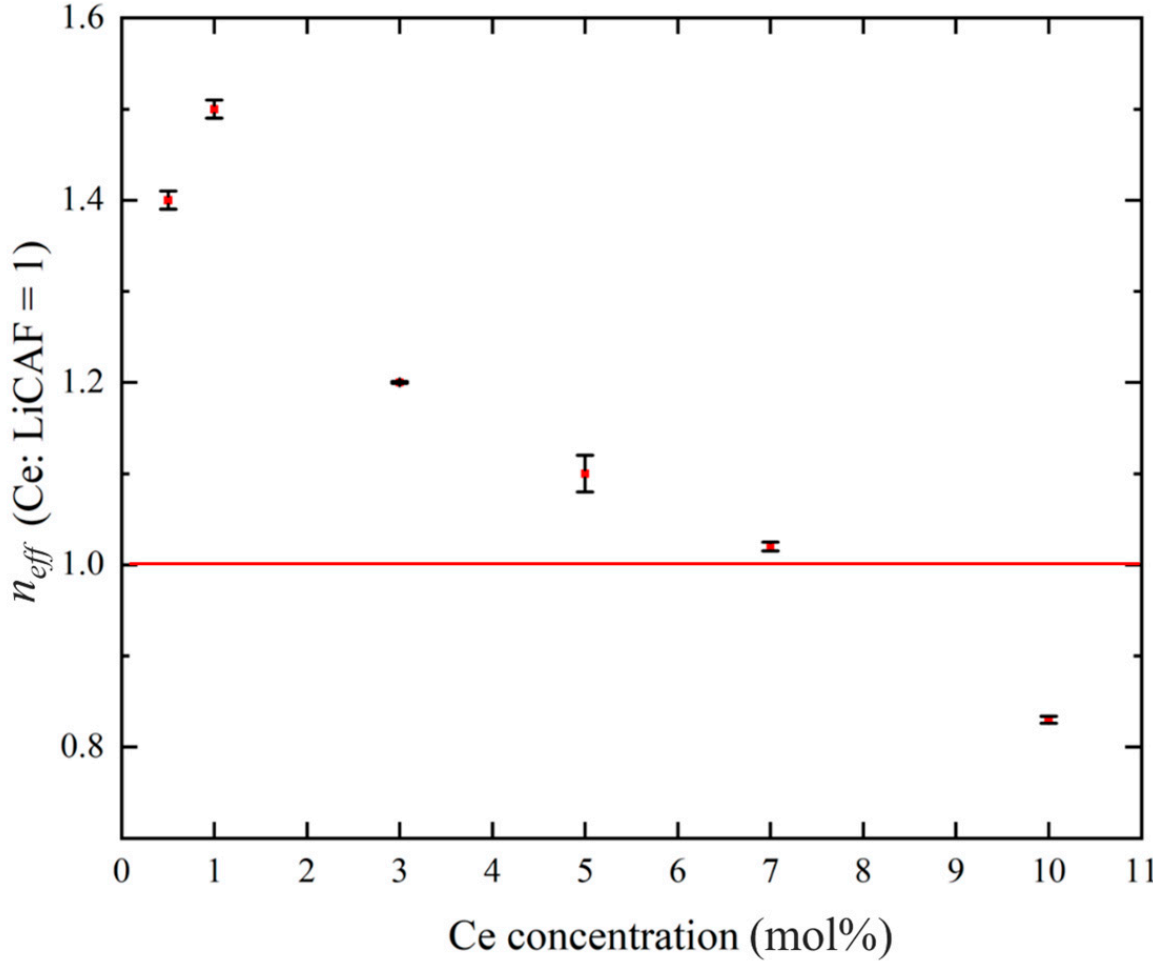
Effective neutron sensitivity plotted against Ce concentrations (0.5, 1, 3, 5, 7, and 10 mol%), normalized with respect to Ce:LiCAF = 1.

Figure 8a–d show the pulse-height spectra (PHS) of the Ce:LiCAF single crystal standard and the 0.5, 5, and 10 mol% Ce-doped eutectic samples under ^252^Cf (neutron) and ^60^Co (γ-ray) irradiation. The Ce:LiCAF standard with a light yield of 5000 ph/n was used as a reference. For all samples, the dead-time ratio (τ_d_) was maintained below 15%, ensuring reliable count-rate correction. The energy resolution of the ^252^Cf-induced peak was 21% for the Ce:LiCAF single crystal, whereas those of the 0.5, 1, 3, 5, 7, and 10 mol% eutectic samples were 21%, 50%, 52%, 79%, 90%, 112%, and 104%, respectively. The degradation in the eutectic samples is primarily attributed to light scattering at grain boundaries inherent to the eutectic microstructure, which induces fluctuations in photon collection efficiency. As shown in the figure, the peak position (μ) of the pulse-height spectra for the Ce:CaF_2_/LiF eutectic obtained under ^252^Cf excitation shifted toward the higher MCA channel side with decreasing Ce concentration, leading to enhanced light yield and neutron sensitivity. The light yields under ^252^Cf irradiation are summarized in Figure 9. The quantum efficiency, corrected for the PMT spectral response, was 0.307 for Ce:LiCAF and 0.390 for the eutectic samples. At low Ce concentrations (0.5–1 mol%), the yields significantly exceeded those of Ce:LiCAF, reaching ~8000 ph/n, about 1.6 times higher than the Ce:LiCAF standard. Subsequently, the effective neutron count rate (n_eff_) was calculated using Equation (4) and normalized to that of Ce:LiCAF (Figure 10). All eutectic samples except the 10 mol% Ce exhibited higher neutron sensitivity than Ce:LiCAF, with the 1 mol% Ce sample achieving the highest value—approximately 1.5 times greater. This enhancement is primarily attributed to the high neutron-induced light yield of Ce:CaF_2_, the low γ-ray light yield, and the high ^6^Li content provided by the eutectic structure.

**Figure 11:**
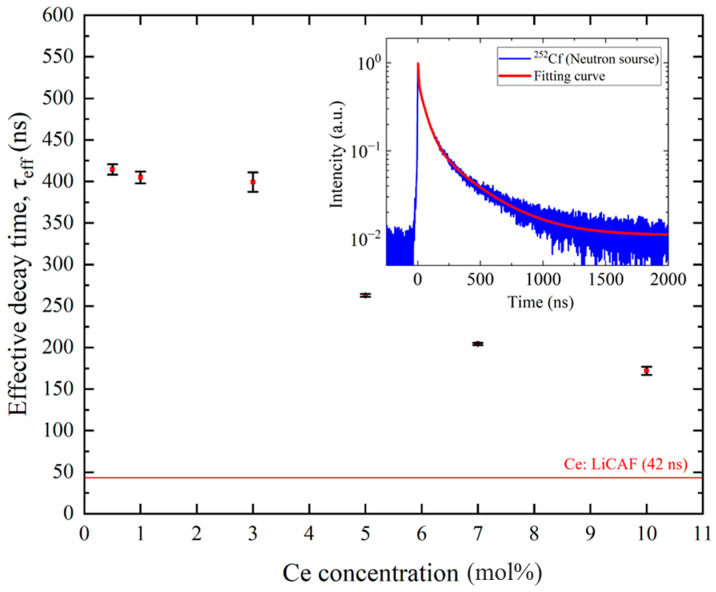
Effective scintillation decay time of Ce:CaF_2_/LiF eutectic crystals under ^252^Cf excitation, plotted as a function of Ce concentration (0.5, 1, 3, 5, 7, and 10 mol%). The inset shows a representative scintillation decay waveform for the 7% Ce-doped sample.

To evaluate the temporal response of the scintillation process, decay-time measurements were performed under ^252^Cf excitation. Ce:LiCAF exhibited a single decay component of 42 ns (100%), corresponding to the Ce^3+^ 5d–4f transition. In contrast, the Ce:CaF_2_/LiF eutectic samples showed two components: 511 ns (75%) and 86 ns (25%) for 0.5 mol% Ce, 520 ns (75%) and 86 ns (25%) for 1 mol% Ce, 534 ns (70%) and 100 ns (30%) for 3 mol% Ce, 389 ns (50%) and 73 ns (50%) for 5 mol% Ce, 321 ns (60%) and 59 ns (40%) for 7 mol% Ce, and 257 ns (60%) and 47 ns (40%) for 10 mol% Ce. The slower components (~250–530 ns) might be ascribed to STE emission, while the faster ones (~50–100 ns) may originate from Ce^3+^ 5d–4f transitions. For the 0.5 mol% Ce sample, the two components—86 ns and 511 ns—aligned with the results reported by Kawano et al. [22]. Figure 11 summarizes the effective decay times (τ_eff_) calculated from these decay components based on Equation (6). The τ_eff_ values were below ~400 ns and decreased with increasing Ce content, reaching a minimum (~170 ns) at 10 mol% Ce. With increasing Ce concentration, the decay time shortened while the light yield decreased. This trend suggests concentration quenching caused by nonradiative energy transfer and cross-relaxation among Ce^3+^ ions. In the CaF_2_/LiF eutectic, however, Ce-rich phases precipitate, limiting the effective Ce^3+^ concentration dissolved in the CaF_2_ phase. As a result, further acceleration of the decay is suppressed, and the ~50 ns fast decay observed in highly doped Ce:CaF_2_ single crystals [16] is therefore difficult to achieve in the present eutectic samples.

Finally, Table 3 summarizes the properties of the conventional Ce:LiCAF single crystal and the 1 mol% Ce:CaF_2_/LiF eutectic, which exhibited the highest effective neutron sensitivity among all samples investigated.

## 4. Conclusions

The optimized composition exhibited the highest effective neutron sensitivity, approximately 1.5 times greater than that of the Ce:LiCAF single-crystal standard, together with a neutron-induced light yield of 7800 ph/n, corresponding to an enhancement of about 1.6 times compared with Ce:LiCAF. At the same time, fast decay components of 86 and 520 ns (τ_eff_ ~ 400 ns) were observed. Furthermore, microstructural analysis—performed for the first time on this eutectic system—revealed the presence of Ce-rich precipitates, namely (Ca_0.68_Ce_0.32_)F_2.32_ and CeF_3_, preferentially distributed along the lamellar CaF_2_/LiF eutectic colony boundaries. At high Ce concentrations, these Ce-rich phases were found to reduce transmittance and limit the effective Ce^3+^ concentration in the CaF_2_/LiF phase, likely hindering the achievement of faster decay and higher light yield. Overall, the Ce:CaF_2_/LiF eutectic scintillator represents a promising alternative to Ce:LiCAF for thermal neutron detection. Its high neutron sensitivity—mainly due to its high Li content (approximately four times that of Ce:LiCAF) and high light yield—combined with sufficiently fast decay, makes it a strong candidate for nuclear decommissioning applications. Future work will focus on controlling the formation of Ce-rich phases at high Ce-doping levels and on clarifying how scintillation decay behavior influences detector performance under even higher radiation-dose environments.

## Figures and Tables

**Table 1:** Summary of the FE-EPMA results, listing the Ca and Ce concentrations (atom%) corresponding to the numbered measurement points indicated in Figure 4. The SD values in the table represent the standard deviation.

No.	Ca	Ce	Total
1	67.89	32.11	100
2	67.24	32.76	100
3	66.69	33.31	100
4	69.57	30.43	100
5	66.36	33.64	100
6	67.76	32.24	100
Mean ± SD	67.59 ± 1.1	32.24 ± 1.1	

**Table 2:** Elemental composition (mol%) of the Ce-doped eutectic with a nominal Ce concentration of approximately 5 mol%, measured by ICP-MS at different vertical positions. The values in parentheses represent the calculated Ce fraction, expressed as 100 × Ce/(Ce + Ca).

	Ce	Ca	Li	Total
Upper part	1.42 (6.14)	21.7	76.9	100
Middle part	1.55 (6.69)	21.6	76.8	100
Bottom part	1.76 (7.53)	21.6	76.6	100

**Table 3:** Summary of the effective neutron sensitivity (n_e_ff, normalized to Ce:LiCAF = 1), light yield (L.Y.), and effective scintillation decay time (τ_e_ff) of prepared Ce:CaF_2_/LiF eutectic crystals under ^252^Cf excitation.

Scintillators	^6^Li Concentration [mol/cm^3^]	Density [g/cm^3^]	Effective Neutron Sensitivity [Ce: LiCAF = 1]	Effective Decay Time [ns]	Light Yield [ph/nth]	Hygroscopicity
Ce: ^6^LiCAF single crystal standard	0.016	3.0	1	40	5000	No
1 mol%Ce:CaF_2_/^6^LiF eutectic (This research)	0.063	2.8	1.5	400	7800	No

## Data Availability

The original contributions presented in this study are included in the article. Further inquiries can be directed to the corresponding authors.
